# Accurate prediction of acute pancreatitis severity with integrative blood molecular measurements

**DOI:** 10.18632/aging.202689

**Published:** 2021-03-10

**Authors:** Hong-Wei Sun, Jing-Yi Lu, Yi-Xin Weng, Hao Chen, Qi-Ye He, Rui Liu, Hui-Ping Li, Jing-Ye Pan, Ke-Qing Shi

**Affiliations:** 1 Department of Hepatobiliary Surgery, The First Affiliated Hospital of Wenzhou Medical University, Wenzhou, Zhejiang Province, China; 2 Translational Medicine Laboratory, The First Affiliated Hospital of Wenzhou Medical University, Wenzhou, Zhejiang Province, China; 3 Singlera Genomics Inc., San Diego, CA 92037, USA; 4 Key Laboratory of Intelligent Critical Care and Life Support Research of Zhejiang Province, Department of Intensive Care, The First Affiliated Hospital of Wenzhou Medical University, Wenzhou, Zhejiang Province, China

**Keywords:** severe acute pancreatitis, venous blood markers, prediction of severity, machine learning

## Abstract

Background: Early diagnosis of severe acute pancreatitis (SAP) is essential to minimize its mortality and improve prognosis. We aimed to develop an accurate and applicable machine learning predictive model based on routine clinical testing results for stratifying acute pancreatitis (AP) severity.

Results: We identified 11 markers predictive of AP severity and trained an AP stratification model called APSAVE, which classified AP cases within 24 hours at an average area under the curve (AUC) of 0.74 +/- 0.04. It was further validated in 568 validation cases, achieving an AUC of 0.73, which is similar to that of Ranson’s criteria (AUC = 0.74) and higher than APACHE II and BISAP (AUC = 0.69 and 0.66, respectively).

Conclusions: We developed and validated a venous blood marker-based AP severity stratification model with higher accuracy and broader applicability, which holds promises for reducing SAP mortality and improving its clinical outcomes.

Materials and Methods: Nine hundred and forty-five AP patients were enrolled into this study. Clinical venous blood tests covering 65 biomarkers were performed on AP patients within 24 hours of admission. An SAP prediction model was built with statistical learning to select biomarkers that are most predictive for AP severity.

## INTRODUCTION

Acute pancreatitis (AP) is one of the most common gastrointestinal emergency conditions [1, 2]. Its clinical severity is stratified into three categories according to Revised Atlanta Classification (RAC): mild, moderately severe, and severe [3]. While both mild (MAP) and severe AP (SAP, including moderately severe and severe cases) patients suffer from pancreatic inflammations, SAP patients are further characterized by failure of one or more organs, and local or systemic complications. Compared to MAP, SAP patients have a much worse prognosis: on average they require significantly longer hospital stay, suffer more frequent complications, and most notably, have a significantly higher mortality rate (up to 20%) [4].

Existing widely used AP severity stratification systems are either imaging-based (for example, Balthazar CT-enhanced scoring system and the computed tomography severity index, or CTSI) [5], or clinical test-based, such as Ranson’s score [6], APACHE-II [7], and BISAP [8], or based on a combination of both clinical tests and patient self-reporting, including pancreatic activity scoring system (PASS) and RAC itself [3, 9]. However, while generally useful, so far all of the aforementioned stratification systems have been shown to predict SAP with a moderate accuracy: for example, two recent studies show that those systems typically achieved an AUC between 0.6 to 0.8 during SAP prediction [10, 11]; additionally, some of those systems demonstrated higher specificity than sensitivity in SAP prediction, or vice versa [12]. Some systems, such as APACHE-II, which requires a total of 16 tests to predict AP severity, are too complicated to perform in typical clinical settings. Some, such as Ranson’s scores, require a minimum of 48 hours to collect all the data points after hospitalization to predict SAP, limiting the time window to initiate medical intervention [13]. Furthermore, imaging-based systems can be influenced by inspectors’ personal experiences [5] when interpreting data. Last but not least, while enhanced CT is essential to detect localized pancreas complications, it may actually complicate treatment by causing deterioration in pancreatic microcirculatory disturbance [14].

Given the limitations of the current AP severity prediction systems, we sought to develop a clinical tests-based scoring model to predict SAP more accurately within the first 24 hours after admission. We focused on blood tests that are routinely performed in hospital, and therefore are practical to implement. We also utilized machine learning techniques to select most-predictive tests and develop prediction models to improve accuracy in stratifying AP severity.

## RESULTS

### Patient characteristics and sample description

We in total collected 945 AP cases admitted to First Affiliated Hospital of Wenzhou Medical University between July 2017 and April 2019. Patients were randomly assigned to a training or a validation cohort at a ratio of 30:70 while maintaining matched age distributions and gender ratios between these 2 cohorts: the 289 AP cases assigned to the training cohort have a median age of 49.2 years (ranging from 20 to 84), and a male-female ratio of 63.1%–36.9%; the 656 AP cases in the validation cohort have a median age of 51.4 years (ranging from 18 to 95), and a male-female ratio of 63.5%–36.5%. Summaries of demographics and clinical features of both cohorts were presented in Table 1.

**Table 1:** Demographics and clinical characteristics, which are included in APSAVE, of the two study cohorts after cases with missing values were filtered out.

	**Training cohort**	**Validation cohort**
**Patient demographics**		
**AP case (*n*)**	234	568
**Median age (range)**	47 (20–84)	50 (18–95)
**Male**	63.1%	63.5%
**Clinical parameters: median (range)**		
**SAP according to RAC (*n*)**	128	162
**SIRS (*n*)**	131	310
**Organ damage (kidney or lung, *n*)**	67	103
**Triglyceride (mmol/L)**	1.665 (0.2–66.62)	1.215 (0.16–76.36)
**Lymphocyte percentage (%)**	0.102 (0.011–0.477)	0.1115 (0.009–0.894)
**Blood urea nitrogen (BUN) (mmol/L)**	4.6 (1.3–22.5)	4.6 (1–34.1)
**Creatinine (μmol/L)**	62 (5–527)	63 (10–996)
**Thrombin time ratio (%)**	0.925 (0.78–1.85)	0.95 (0.74–3.45)
**Prothrombin time (s)**	13.7 (11.4–26.8)	13.7 (10.8–49.5)
**Serum potassium (mmol/L)**	3.815 (2.68–5.38)	3.91 (2.68–6.29)
**Plateletcrit (L/L)**	0.23 (0.06–0.55)	0.23 (0.02–0.9)

For all patients, on day 1 of their hospital admission, body fluids samples (venous blood, arterial blood and urine) were collected for clinical tests.

### Identify venous blood markers and build a stratification model to assess AP severity during the first 24 hours of admission

**Figure 1:**
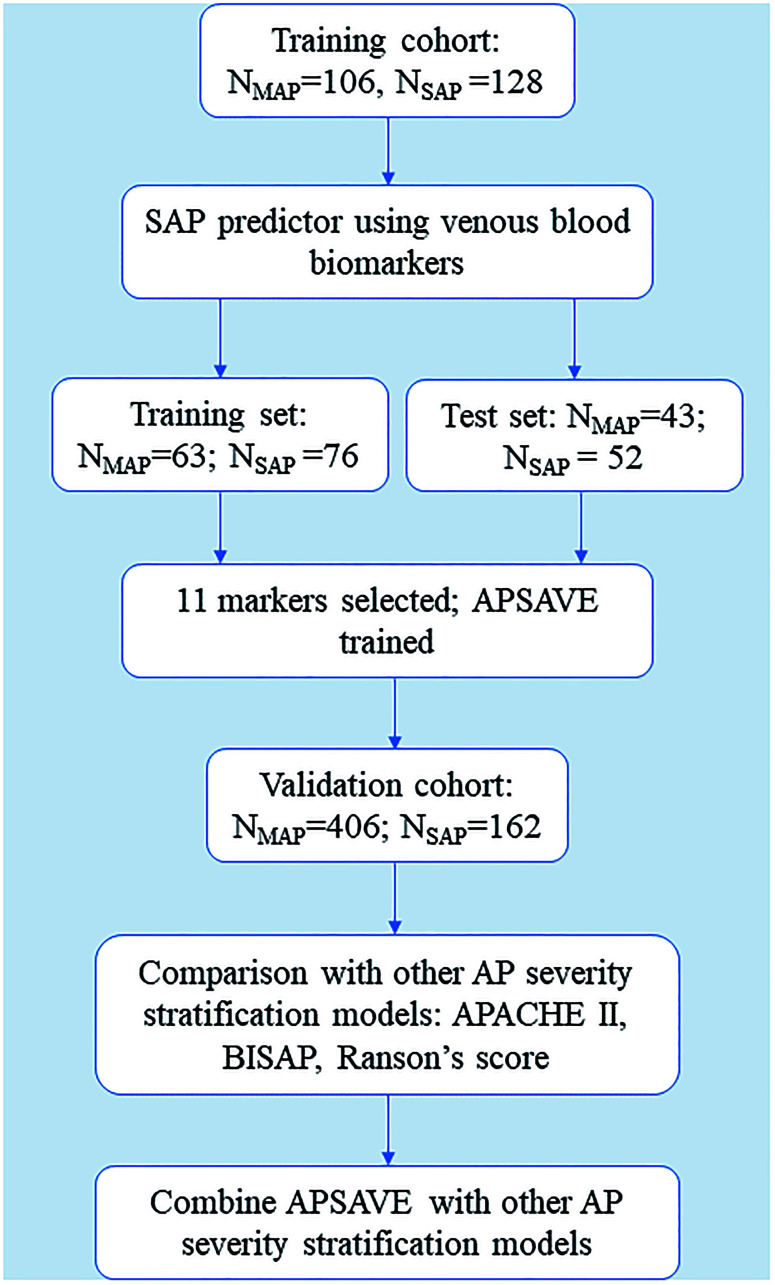
**Flowchart of the training and validation of APSAVE, an AP severity stratification model based on venous blood biomarkers.**

Our analyses process includes biomarker discovery, model training and validation (summarized in Figure 1). For marker discovery, we surveyed the results of 92 non-invasive clinical tests that were performed on our study cohorts, 65 of which were tests on venous blood, 17 on arterial blood and 10 on urine samples (for a full list, see Supplementary Table 1). We also included body temperature in analyses. For benchmarking the performance of an AP stratification system, we developed based on those tests, each case was first classified by RAC for its severity.

We performed a proof-of-principle prediction of SAP cases using all the available clinical test results on a small subset of AP cases in the cohort using the Random Forest method, and achieved a reasonably high accuracy (data not shown). We thus reasoned that by excluding less SAP-predictive tests and rebuilding the AP severity stratification models using only highly predictive tests, we may further improve the models’ accuracy and at the same time reduce models’ complexity. We also predicted that the highly predictive tests should be more indicative of the pathologies of SAP, such as organ damages, complications and/or systematical inflammatory responses.

We focused on the 65 tests that measure biomarkers in venous blood because a venous blood sample is relatively safe and easy to collect from an AP patient. Also, the venous blood tests we selected for the initial screening can have their results returned within the first 24 hours after blood was drawn, thus providing an optimal time window of AP classification.

We filtered the 289 AP cases of the training cohort based on the availability of the 65 blood test results, and left with 234 cases. According to their RAC grades, this cohort consists of 106 MAP cases and 128 SAP cases (55 moderately severe AP cases, and 73 severe AP cases).

During marker discovery and model building for AP severity stratification, we chose to combine moderately severe and severe AP cases into one group instead of treating them as two separate groups. This is because we aimed to maximize the sensitivity of our model to predict SAP cases to prevent further deterioration for even moderately severe AP cases. We reasoned our approach should increase the likelihood of identifying biomarkers highly indicative of SAP core pathologies.

The training cohort was randomly split into a training set and a test set at a ratio of 60:40. Data normalization was performed on all test results using python package “StandardScaler”. The model building process has three main steps: in step 1 we applied Recursive Feature Elimination algorithm (Python package “RFE”) on the training set to identify an optimal number of features based on their importance; in step 2, we built a logistic regression model using the identified features; finally, in step 3, we calculated he AUC of the logistic regression model in classifying the training set as a preliminary validation.

**Figure 2:**
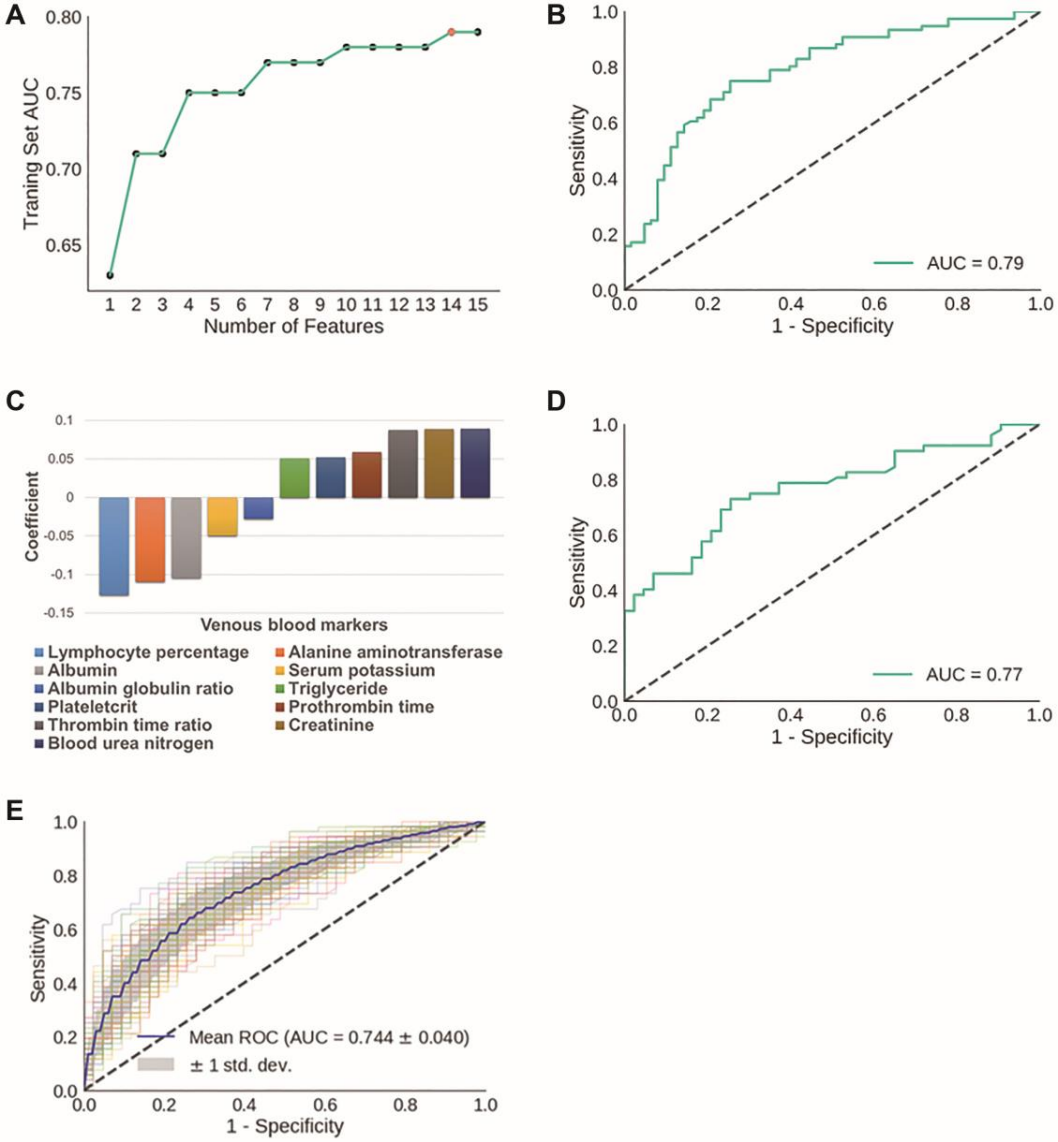
(**A**) Dynamic changes of AUC scores in classifying training set AP cases with gradually decreased number of features. The 14 features identified in initial biomarker screening are: mean hemoglobin level, albumin-globulin ratio, alanine aminotransferase, thrombin time ratio, plateletcrit, albumin, prothrombin time, creatinine, serum potassium, BUN, prothrombin activity, mean hemoglobin concentration, triglyceride, lymph percentage. (**B)** ROC curve for the classification of AP training set; (**C**) Components of APSAVE and their corresponding coefficients; (**D**) ROC curve for the classification of AP test set; (**E**) Average ROC curve when training set and test set samples were reshuffled 100 times.

In practice, in step 1 we repeatedly ran RFE with a preset feature number ranging from 1 to 15. Feature numbers greater than 15 were not considered to minimize the possibility of overfitting classification models and detracting from the simplicity and applicability of our models. We reached the highest AUC at 0.79 (sensitivity: 72.4%; specificity: 71.4%) with 14 features (Figure 2A).

To further minimize the chance of model overfitting without compromising its accuracy, we exhaustively eliminated individual features one by one manually from the 14-feature model, and re-calculated the AUC scores of each new prediction model. We found that we were able to maintain the AUC score at 0.79 (sensitivity: 73.7%; specificity: 74.6%) after eliminating 3 features: mean hemoglobin quantity, mean hemoglobin concentration and prothrombin activity (Supplementary Table 2). Further elimination of any of the remaining 11 tests caused significant decrease in the accuracy of SAP-prediction. We then built a logistic regression model using the remaining 11 features/biomarkers to stratify AP cases based on severity (Figure 2B–2C), which we termed Acute Pancreatitis Stratification using Venous blood (APSAVE). APSAVE will calculate a score to each AP case based on the 11 tests’ results, and the threshold score was set at 0.5 to discriminate SAP from MAP cases: AP cases scored equal or above 0.5 are classified as SAP, while those scored below 0.5 are MAP cases.

We predicted 95 AP cases’ severity in the test set for a preliminary validation of APSAVE, and it achieved an AUC of 0.77 (sensitivity: 71.2%; specificity: 74.4%) (Figure 2D). To demonstrate the robustness of APSAVE, we randomly shuffled training/test splits 100 times and generated an average ROC curve for validations (Figure 2E), which has an average AUC of 0.74 ± 0.04, demonstrating its consistency in SAP prediction.

### APSAVE demonstrated a high degree of accuracy in classifying validation AP cases in a single-blinded manner

**Figure 3:**
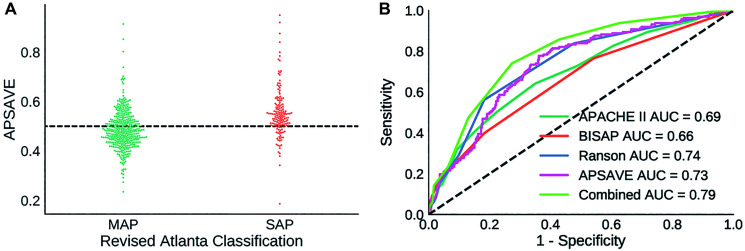
(**A**) Distribution of APSAVE scores of MAP and SAP cases in the validation cohort; the dashed black line indicates the numeric cutoff of 0.5 to classify an AP case as mild or severe. (**B**) AUC curves of APSAVE, APACHE II, BISAP, Ranson’s criteria and the combined model (“Combined”) of APSAVE + Ranson’s on classifying AP cases of the validation cohort.

We performed a single-blinded prediction of AP cases to validate APSAVE. The validation cohort has 656 AP cases collected from July 2017 to April 2019. Cases missing value for any of the 11 biomarkers were left out, which resulted in 568 cases for validation (Table 1). According to RAC, this cohort consists of 406 MAP cases and 162 SAP cases (84 moderately severe AP cases, and 78 severe AP cases). Data normalization was performed in the same way as in model building. We applied APSAVE to the validation cohort to predicted SAP cases and compared the prediction results with their RAC grades, which showed a sensitivity of 78.4% and a specificity of 61.8% (an AUC = 0.73, Figure 3), which was similar to the results in the training cohort.

### APSAVE is highly accurate and more sensitive in detecting SAP during its early phase than other AP stratification systems

To further assess the accuracy of APSAVE, we classified the validation cohort using 3 clinically frequently used SAP prediction systems: APACHE II, BISAP, and Ranson’s Criteria, and compared their classification results with those of APSAVE.

It should be noted that APSAVE shares several markers with those 3 systems. Both APSAVE and APACHE II measure potassium and creatine levels in serum; similarly, APSAVE, BISAP and Ranson’s Criteria all measure blood urea nitrogen (BUN) in blood. Such agreements indicate APSAVE may have captured dynamical changes of metabolites in blood that are highly indicative of AP pathology.

All 568 AP cases from the validation cohort contain complete data required for those 3 classification systems. Therefore, scores for those systems were calculated without imputation and were used to classify AP cases in accordance with their individual formula [6, 8, 15]. The ROC curves of those systems were shown in Figure 3B, and their sensitivity, specificity and AUC values were summarized in Table 2.

**Table 2:** AUC, sensitivity and specificity in classifying validation AP cases by APSAVE, APACHE II, BISAP, Ranson’s criteria and the combined model of APSAVE and Ranson’s (“Combined”).

**Model/System**	**AUC**	**Sensitivity**	**Specificity**
**APACHE II**	0.69	42.0%	84.0%
**BISAP**	0.66	40.1%	81.5%
**Ranson**	0.74	56.2%	81.8%
**APSAVE**	0.73	78.4%	61.8%
**Combined**	0.79	74.1%	72.7%

Overall APSAVE and Ranson’s Criteria have the highest degree of accuracy (AUC = 0.73 and 0.74, respectively, Figure 3B and Table 2) in classifying the validation cohort, performing better than APACHE II and BISAP (AUC = 0.69 and 0.66, respectively). Furthermore, APSAVE has a higher degree of sensitivity in predicting SAP cases than all other 3 systems (Table 2), suggesting that APSAVE is more likely to detect SAP cases in their early stage than the other 3 systems.

### The combined model of APSAVE and Ranson’s criteria had an increased accuracy in classifying AP cases on the validation cohort

Given that APSAVE is more sensitive but less specific in classifying SAP cases compared to the 3 established AP stratification systems, we reasoned that it is possible to further improve the accuracy of AP stratification by integrating APSAVE with one of the other 3 systems. We explored this possibility by combining the score of APSAVE with that of APACHE II, BISAP or Ranson’s criteria, respectively and used each aggregate score to classify the validation cohort. We found that each aggregate score showed improvement over its parental score alone (Supplementary Figure 1). Among them, the aggregate score formulated by adding a Ranson’s criteria’s score of an AP case to twice of its APSAVE score has the highest overall accuracy on the validation cohort: indeed, it substantially improved APSAVE’s specificity at a relatively small tradeoff of its sensitivity (sensitivity 74.1%, specificity 72.7%, AUC = 0.79, Table 2).

## DISCUSSION

Early detection of SAP remains a challenge in the emergency care of AP patients, and is key to SAP patients’ immediate survival and long-term prognosis. RAC, being the gold standards of AP diagnosis, requires more than 48 hours to assess the severity of an AP case, which limits its utility. Other diagnostic protocols either require more than 48 hours to have results returned, or are challenging to perform and score.

We pursued a strategy by unbiasedly identifying clinical tests on body fluids whose results collectively can accurately stratify AP’s severity. By perusing over 90 different tests and applying machine-learning algorithms in feature selection and modeling, we selected 11 tests and built APSAVE, an AP severity stratification model using the 11 tests’ measurements to diagnose SAP cases during the first 24 hours of admission. Using retrospective AP cases from a single center for validation, their RAC classification results as gold standards, and 3 widely used AP stratification systems for comparisons, we demonstrated that APSAVE has a comparable accuracy as the Ranson’s criteria in diagnosing SAP, and outperformed APACHE II and BISAP, confirming APSAVE’s accuracy and robustness. Notably, our system has a much higher sensitivity than the other 3 systems to diagnose SAP, which may be advantageous for SAP’s timely treatment to improve its prognosis, especially to reduce its mortality rate. Also, our system uses the same tests for all AP cases regardless of whether they were caused by gallbladder stones or not, which simplifies the procedure.

The 11 clinical tests included in APSAVE were performed on venous blood, which is relatively safe and easy to be collected from AP patients. These tests are routinely performed in hospitals, even in primary care settings, which may reduce the cost to implement our system. While these clinical tests may need to be performed in different laboratories in a hospital, with recent advancements in electronic medical recording and data transmission, test results collection and AP severity scoring can be automated and easily accessible to physicians caring AP patients, thus streamlining the diagnosis process.

Among the 11 tests that consist of APSAVE, we have independently identified several that also have been included in other classification systems: BUN (BISAP and Ranson’s Criteria), creatinine level (APACHE II), serum potassium (APACHE II) and Albumin (Glasgow pancreatitis score [16]). These agreements demonstrate APSAVE captured the same key features of AP pathologies, such as kidney damages and inflammatory responses [17], as other systems did, supporting the validity of our approach and identified biomarkers.

We also found that APSAVE is complementary to Ranson’s in AP classification: we combined scores between APSAVE and each of the other three system to predict AP severity, and found that the aggregate score from APSAVE and Ranson’s criteria predicted SAP cases substantially more accurately overall than either score alone, and better than the aggregate scores consisting APSAVE and APACHE II or BISAP, respectively. This indicates potential benefits in building a combined SAP classification system using APSAVE and Ranson’s criteria. However, it should be noted that to apply such a model clinically, more blood samples and tests are required for each AP patient; how such requirements affect its clinical applicability and affordability needs further investigations to address.

Besides markers shared between APSAVE and other established AP stratification systems described above, several other biomarkers in APSAVE have also previously been proposed for AP classification. The blood level of alanine aminotransferase (ALT) has been shown to be highly positively predictive for gallbladder stone-caused AP [15], thus as a marker to classify AP into subtypes based on etiology. In our study, we found ALT level is higher on average in MAP than in SAP cases (Supplementary Figure 2), suggesting that etiology may be a factor in the severity of AP.

Coagulation abnormalities, including intravascular thrombosis to disseminated intravascular coagulation (DIC) [18, 19], were caused by system inflammatory response syndrome (SIRS) during AP episodes (for review, see Kakafika et al. [20]). Indeed, prothrombin time was reported to be longer in AP cases, especially in SAP [18, 21]. Here, for the first time, we propose that additional coagulation-related metrics, such as plateletcrit and thrombin time, are also quantitative biomarkers to assess AP severity. We speculate that together with lymphocyte percentage, these biomarkers may monitor SIRS induced by pancreas damages.

There are several limitations on APSAVE. First, it was developed using retrospective AP cases from a single hospital, which limits sizes of study cohorts, and their demographic and the behavioral diversity. Significant ethnical and/or regional differences in clinical AP cases’ etiology and epidemiology have been reported globally and within China [22–25], some of which may be attributed to risk factors such as genetics, environment and lifestyle (e.g., smoking, alcohol consumption, etc.), etc. Collaborations with additional hospitals on prospective patients are needed to further validate and refine APSAVE to improve its applicability on diverse populations.

Additionally, while it has a higher sensitivity than the compared existing systems, APSAVE is admittedly less specific, which may lead to overtreatment of MAP cases. By analyzing false-positive cases classified by APSAVE (i.e., MAP cases mis-classified as SAP), we found that the false-positive cases as a group tend to be scored higher than the true-negative cases by APACHE-II, BISAP or Ranson’s Criteria too (Man-Whitney test, *p* < 0.0001 for APACHE II and Ranson’s Criteria, and *p* = 0.0092 for BISAP, Supplementary Table 3 and Supplementary Figure 3). This indicates that broadly speaking, those false-positive cases are the more severe MAP cases. In addition, as to the false-positive outliers, we found that they have more similar test results as the true positives than the false-positive non-outliers do in majority of the 11 APSAVE tests (Supplementary Figure 4A, 4B), which suggested that those outliers, which were MAP cases, do have key clinical symptoms similar to genuine SAP cases.

We further reviewed the comorbidities of the validation cohort, and found that the percentage of false-positive cases that also have SIRS is significantly higher than that of the true-positive cases (*p* < 0.0001, chi-square test). We speculate that SIRS may have been one of underlying pathologies that have caused those cases to be scored higher than average MAP cases, because SIRS not only enhances inflammatory responses, but also activates coagulation pathways, both of which increase APSAVE scores.

The current numeric cutoff of APSAVE for SAP is set at 0.5 to balance both specificity and sensitivity. Indeed, when we increased the cutoff to 0.54, the APSAVE classified the validation cohort to a specificity of 80.3% and a sensitivity of 49.3%, which is comparable or even slightly better than APACHE-II or BISAP (date not shown), but the reduction of sensitivity from 78% to 49% is substantial. Given the relatively small size of our single-center study cohort, the APSAVE’ cutoff value and the coefficients of its individual tests can and should be further optimized by applying it on substantially larger and more diverse cohorts of AP patients in future studies.

In conclusion, we have demonstrated that APSAVE, an AP stratification system consisting of 11 venous-blood tests, has a high degree of accuracy, consistency, simplicity and applicability on predicting clinical SAP cases, and is highly sensitive to diagnose early-stage SAP cases. An integrated AP scoring system can be developed based on our model to improve the care and management of AP patients.

## MATERIALS AND METHODS

### Study design and participants

This was a retrospective study that was based on a case-control design. Its participants were from AP patients prospectively recruited between July 2017 and April 2019 by the Pancreatitis Unit of the First Affiliated Hospital of Wenzhou Medical University, a university-affiliated tertiary-care public hospital. The study was preformed according to Standards for the Reporting of Diagnostic Accuracy Studies guidance for observational studies. The research protocol of the study was approved by the Ethics Committee of the First Affiliated Hospital of Wenzhou Medical University (2017-136) and written informed consent was obtained from each patient or their next of kin included in the study. The study was registered in Chinese Clinical Trial Registry (ChiCTR-DDD-17012200)

AP was defined as two or more of the following conditions: characteristic abdominal pain; serum amylase and/or lipase levels three or more times of the upper limit of normal; and/or an imaging study (computed tomography (CT) or magnetic resonance imaging) demonstrating changes consistent with AP. Inclusion criteria were: the first episode of AP as defined by the revised Atlanta classification; 18 years and older; male or female; and availability of blood samples within 24 h of admission. Patients were excluded with following criteria: advanced pulmonary, cardiac, renal diseases (chronic kidney disease stage 4–5), liver cirrhosis (Child–Pugh grade B-C) or malignancy; pregnancy; chronic pancreatitis or trauma as the etiology; nonpancreatic infection or sepsis caused by a second disease; the duration of abdominal pain before admission exceeds 24 h. The severity of AP was stratified as mild AP or moderately severe/severe AP according to revised 2012 Atlanta criteria [3].

The primary objective of this study was to identify predictive blood markers for SAP diagnosis and use them to establish an SAP classification system that was both accurate and applicable in a standard clinical setting. For this purpose, the new model was compared with the existing systems being run in clinics, including Ranson’s score, APACHE-II and BISAP.

The demographic, clinical, and laboratory data of all patients with AP at the first day of admission was prospectively collected and maintained in an electronic database in accordance with the protocol for this study, including age, gender, vital signs, physical exam findings, and all available clinical test results. All clinical tests were performed according to standard protocols.

### AP classification modeling

All recruited AP cases were filtered based on availability of test results, then divided into training and test sets for model building and validation, respectively. We first built an SAP prediction model with all available biomarkers using Random Forest algorithm. For the SAP model using only venous blood biomarkers, we implemented Recursive Feature Elimination algorithm (Python package “RFE”) to identify a preset number of tests whose results classified MAP and SAP samples with the highest accuracy in the training set, and further reduced the number of required tests until the AUC of classifying the training set began to decrease. We used Python package “LogisticRegression” to build the APSAVE prediction model.

### Validation

To demonstrate the accuracy and robustness of APSAVE, we performed further validation on the validation set in a single-blinded manner, and compared its performance with three most widely used AP stratification systems: APACHE-II, BISAP and Ranson’ criteria.

To test whether APSAVE can be further improved for accuracy, we integrated APSAVE with APACHE II, BISAP or Ranson’s criteria, respectively, to re-classify the validation set, and compared the SAP-prediction accuracy of the integrated systems with that by the parental systems alone. The codes for model building and validation were listed in.

## Supplementary Material

Supplementary Figures

Supplementary Tables
