# Supplementary material for: Accurate prediction of acute pancreatitis severity with integrative blood molecular measurements
Source: Aging (Albany NY). 2021 Mar 10;13(6):8817–34. doi: 10.18632/aging.202689 (PMC8034948; doi:10.18632/aging.202689)
Supplement: Supplementary Figures [file aging-13-202689-s001.pdf]

SUPPLEMENTARY FIGURES

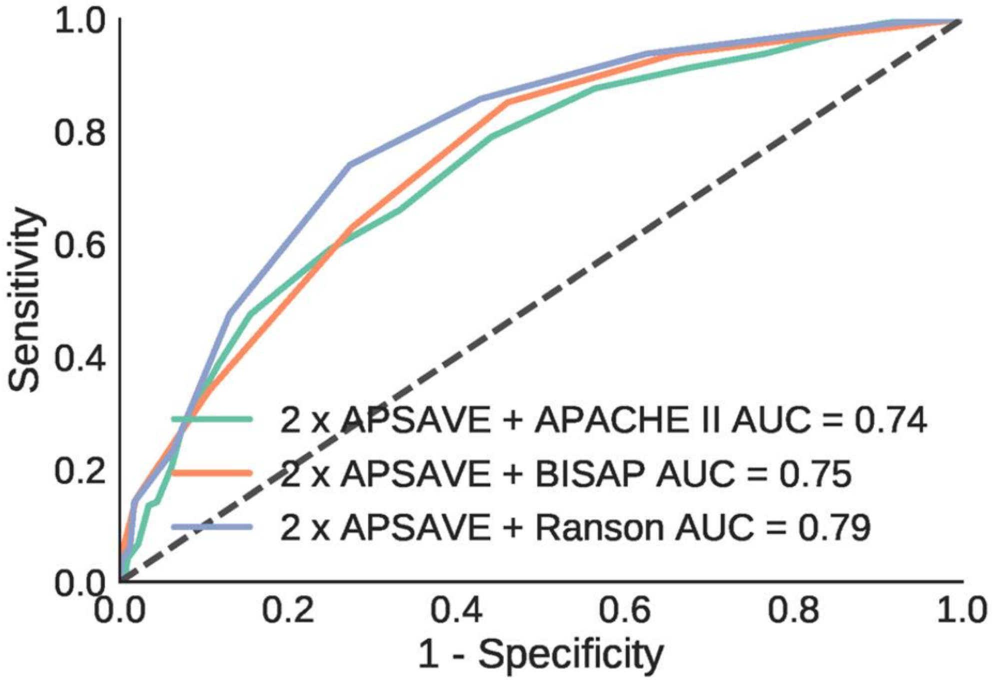

Supplementary Figure 1. AUC curves of combinatorial models of APSAVE plus APACHE II, BISAP or Ranson's criteria classifying AP cases of the validation cohort.

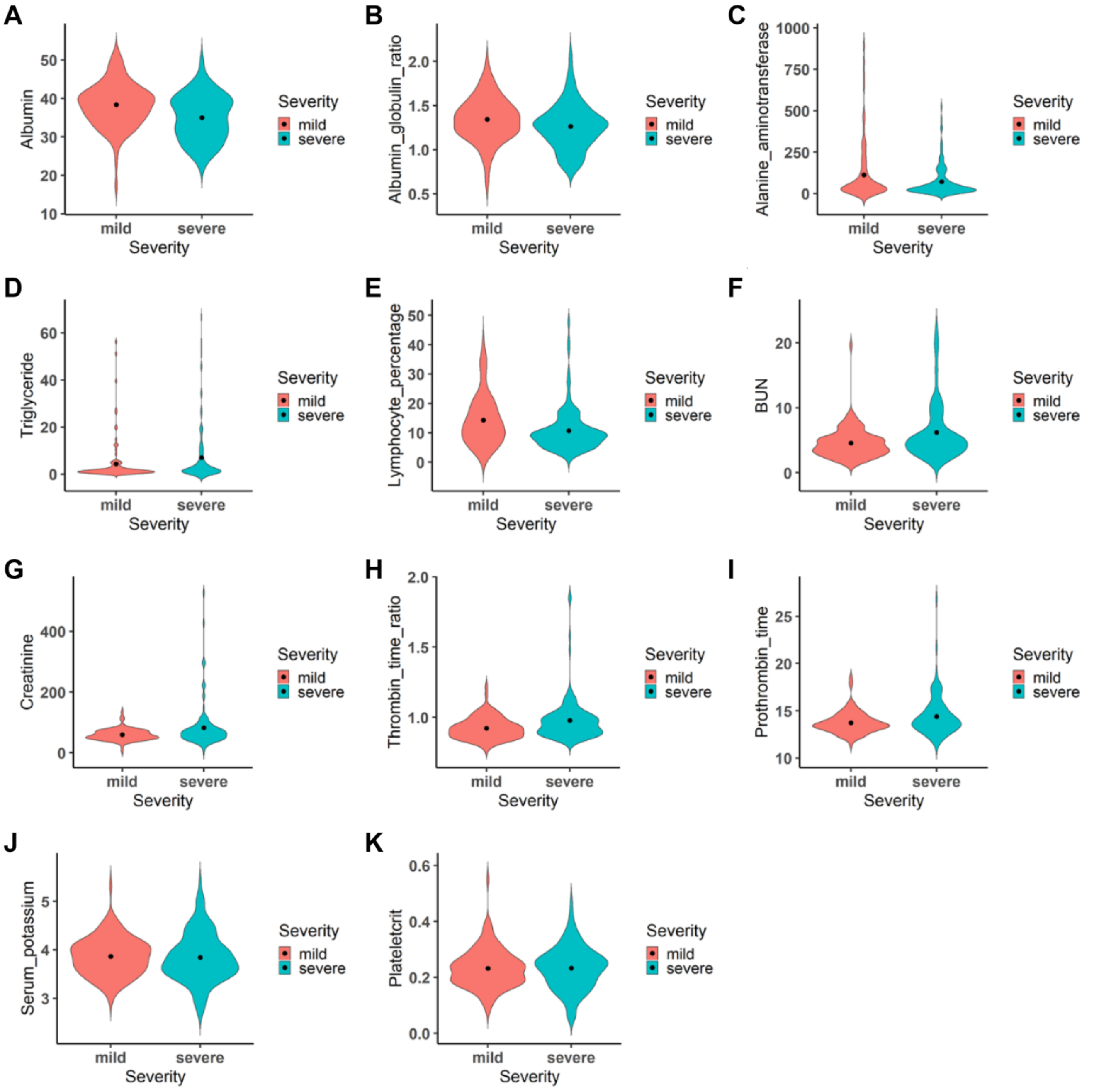

Supplementary Figure 2. Distributions of test results of the 11 biomarkers consisting the APSAVE model from the validation cohort are visualized in violin plots.

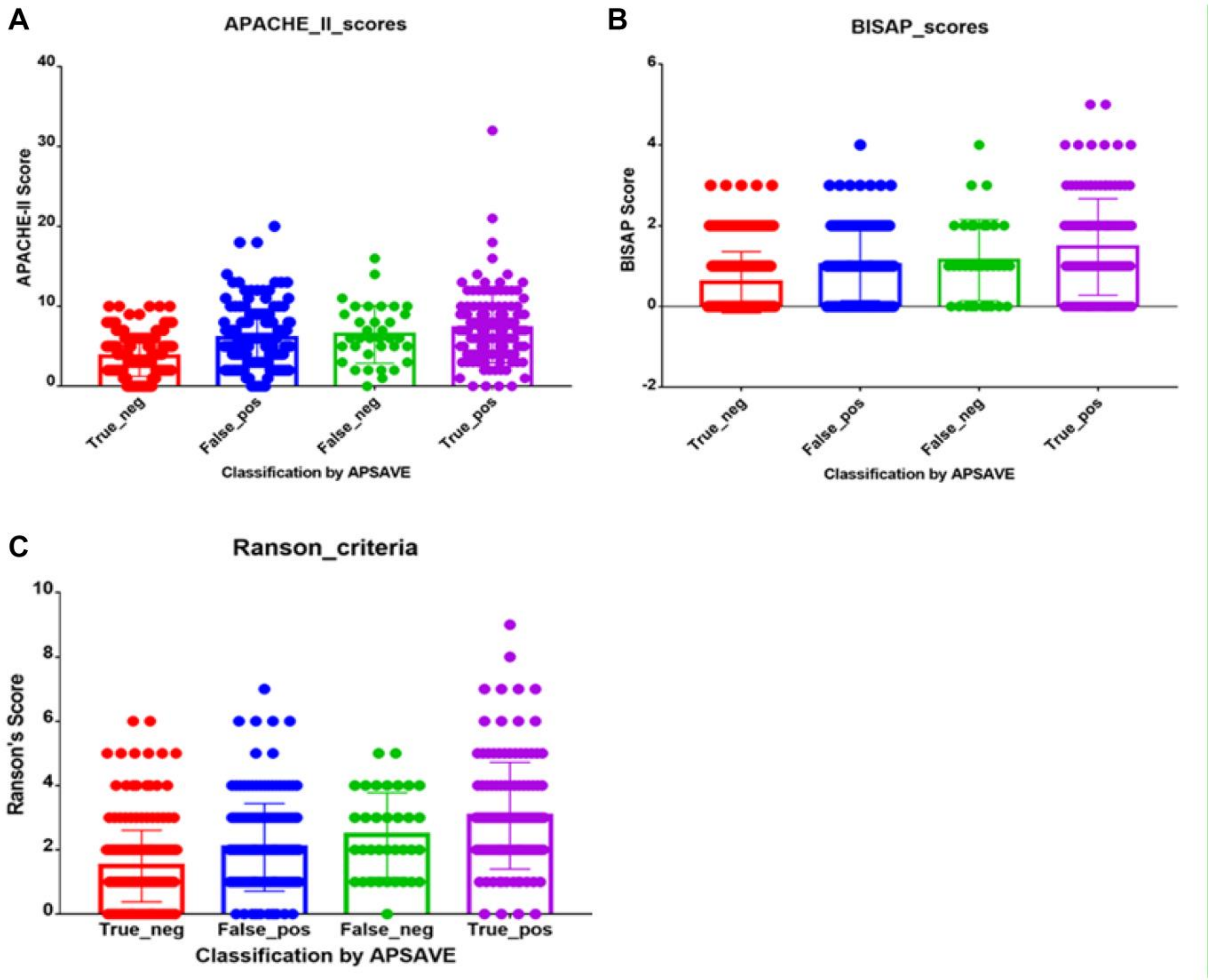

Supplementary Figure 3. False_neg cases (MAP cases mis-classified by APSAVE as SAP) were generally scored higher by either of the 3 stratification systems than the Ture_neg cases (Man-Whitney test, $p < 0.0001$ for APACHE II and Ranson's Criteria, and $p = 0.0092$ for BISAP).

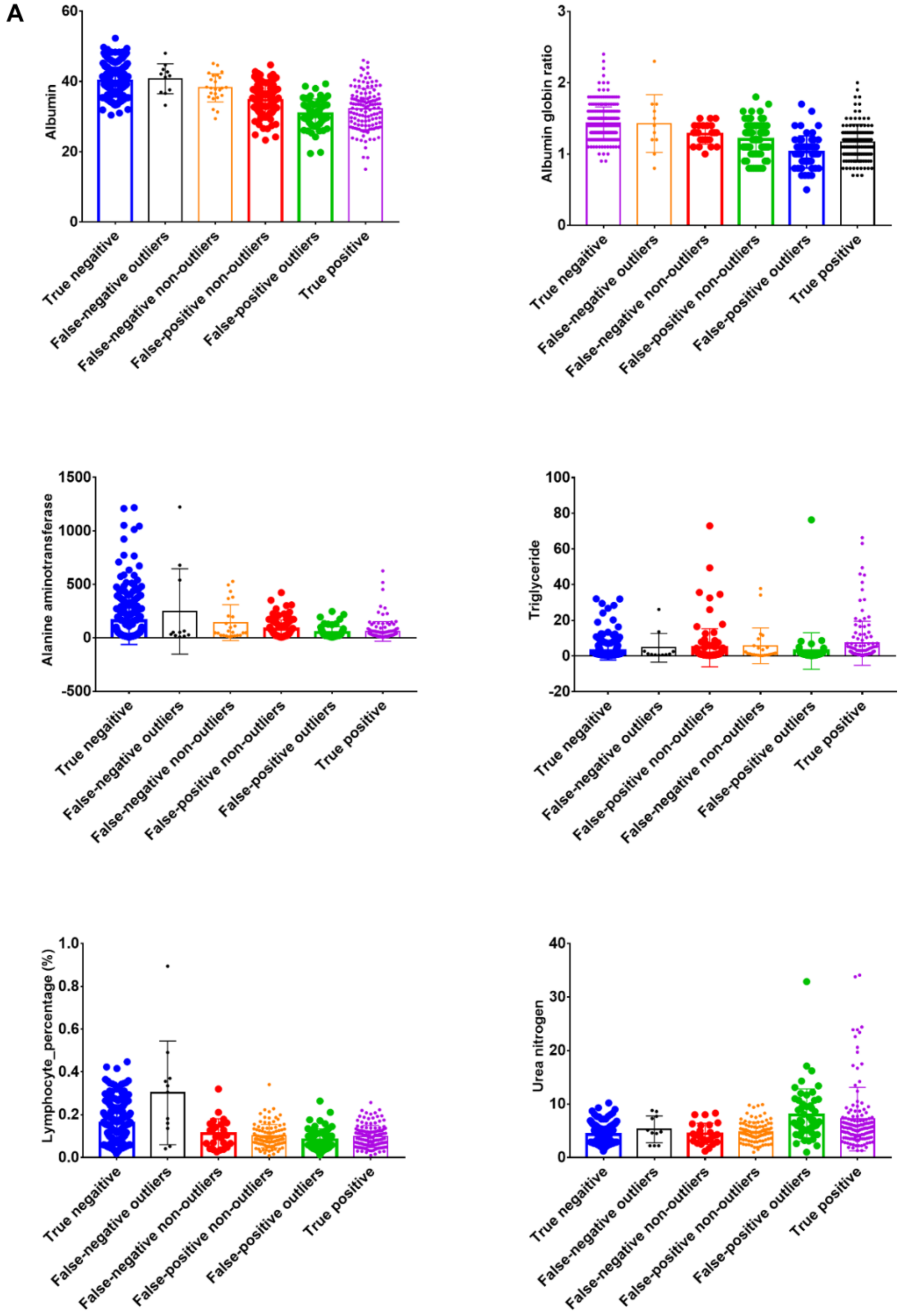

Supplementary Figure 4A. The case distributions of true negative, false-negative outliers, false-negative non-outliers, false-positive non-outliers, false-positive outliers and true positive of 11 biomarkers in the APSAVE model.

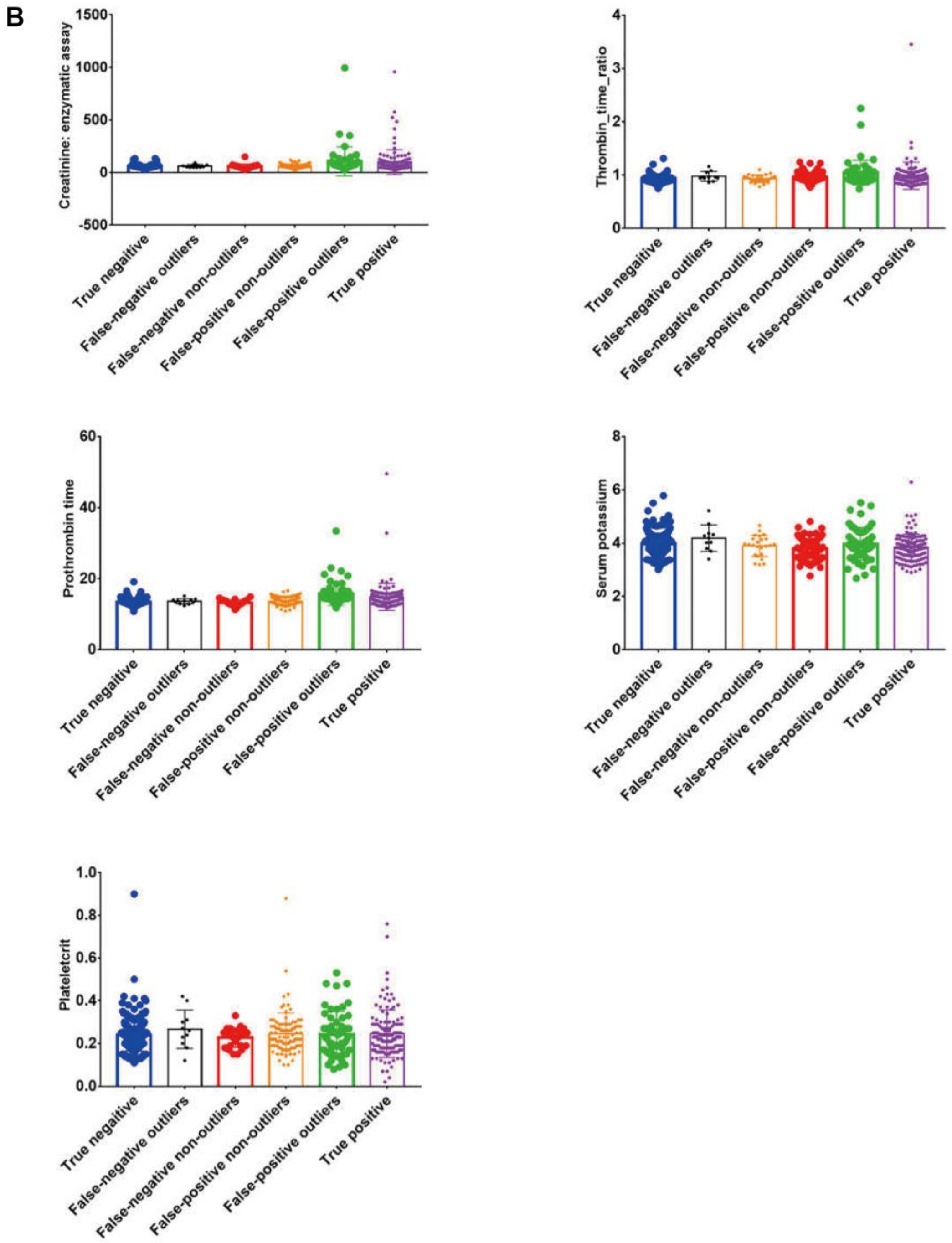

Supplementary Figure 4B. The case distributions of true negative, false-negative outliers, false-negative non-outliers, false-positive non-outliers, false-positive outliers and true positive of 11 biomarkers in the APSAVE model.
