# Supplementary material for: Accurate prediction of acute pancreatitis severity with integrative blood molecular measurements
Source: Aging (Albany NY). 2021 Mar 10;13(6):8817–34. doi: 10.18632/aging.202689 (PMC8034948; doi:10.18632/aging.202689)
Supplement: Supplementary Tables [file aging-13-202689-s002.pdf]

SUPPLEMENTARY TABLES

Supplementary Table 1. List of 93 biomarkers and vital signs which were measured/collected from AP patients of study cohorts used in this study.

Biomarker	Body fluid
Body temperature	Vital sign
HCO3-（Bicarbonate）	Arterial blood
Blood pH	
Buffer base	
Arterial carbon dioxide partial pressure ($PaCO_2$)	
Total carbon dioxide	
Fraction of inspiration Oxygen (FIO2)	
Hydrogen ion concentration	
Oxygen concentration	
Arterial oxygen partial pressure (PaO_2)	
Arterial oxygen saturation（SaO_2）	
pH (temperature)	
PO2 (temperature)	
Pulmonary arterial oxygen partial pressure	
Pulmonary arterial oxygen partial pressure differential	
Base Excess	
Standard bicarbonate	
Standard pH	
Crystal by IQ200	Urine
Mucus by IQ200	
Squamous epithelia by IQ200	
Tube type by IQ200	
Urea creatinine ratio	
Urine amylase	
Urine Specific Gravity	
Urine pH	
White blood cell counts	
White blood cell counts by IQ200	
Activated partial thromboplastin	Venous blood
Alanine aminotransferase	
Albumin	
Albumin/globulin ratio	
Alanine aminotransferase / Aspertate aminotransferase ratio	
Apolipoprotein AI	
APTT ratio	
Aspartate aminotransferase	
Basophil (absolute count)	
Basophil (percentage)	

Blood urea nitrogen
Blood urea nitrogen (Emergency room measurement)
Indirect bilirubin
Total bilirubin
C peptide level after fasting
C reactive protein
Total cholesterol
Creatinine
Creatinine (emergency room)
Estimated glomerular filtration rate eGFR
Eosinophil (absolute count)
Eosinophil percentage
Fibrinogen
Globulin
Glucose
Glucose (Emergency room measurement)
High density lipoprotein cholesterol
Hematocrit
Insulin during fasting
Large platelet ratio
Low density lipoprotein cholesterol
Lipoprotein (a)
Lymphocyte (absolute count)
Lymphocyte (percentage)
Mean hemoglobin
Mean hemoglobin concentration
Mean platelet volume
Mean RBC volume
Monocyte (absolute count)
Monocytes (percentage)
Neutrophil (absolute count)
Neutrophil (percentage)
Platelet
Platelet distribution
Plateletcrit
Protein total
Prothrombin activity
Prothrombin time
Red blood cell volume distribution
Red blood cell volume distribution width
Red blood cell
Red blood cell by IQ200
Serum amylase
Serum amylase (Emergency room measurement)

Serum chloride
Serum chloride (Emergency room measurement)
Serum potassium
Serum potassium (Emergency room measurement)
Serum sodium
Serum sodium (Emergency room measurement)
Thrombin time
Thrombin time ratio
Triglyceride
Uric acid

Supplementary Table 2. 14 clinical tests were selected by RFE algorithm based on feature importance; 3 of them were further eliminated to obtain the minimal set of features while maintaining accuracy for classification.

Clinical Test	Selected by RFE	Kept in APSAVE
Mean hemoglobin	Yes	No
Albumin globulin ratio	Yes	Yes
Alanine aminotransferase	Yes	Yes
Thrombin time ratio	Yes	Yes
Plateletcrit	Yes	Yes
Albumin	Yes	Yes
Prothrombin time	Yes	Yes
Creatinine	Yes	Yes
Serum potassium	Yes	Yes
Blood urea nitrogen (BUN)	Yes	Yes
Prothrombin activity	Yes	No
Mean hemoglobin concentration	Yes	No
Triglyceride	Yes	Yes
Lymph percentage	Yes	Yes

Supplementary Table 3. Numbers of true negatives, false positives, true positives and false negatives predicted by APACHE II, BISAP, Ranson's criteria, and APSAVE on validation cohort using RAC grades as the gold standard.

	RAC	APACHE II	BISAP	Ranson	APSAVE	
Mild	406	341	331	332	251	True negative
		65	75	74	155	False positive
Severe	162	68	65	91	127	True positive
		94	97	71	35	False negative
